# Adult Head and Neck Soft Tissue Sarcomas: Treatment and Outcome

**DOI:** 10.1155/2008/654987

**Published:** 2008-03-18

**Authors:** Rabindra P. Singh, Robert J. Grimer, Nabina Bhujel, Simon R. Carter, Roger M. Tillman, Adesegun Abudu

**Affiliations:** ^1^King's College Hospital, Denmark Hill, London SE5 9RS, UK; ^2^Royal Orthopaedic Hospital, Bristol Road South, Birmingham B31 2AP, UK; ^3^Oral and Maxillofacial Surgery, University Hospitals Birmingham, Birmingham B15 2TH, UK

## Abstract

We have retrospectively analysed the experience of a musculoskeletal oncological unit in the management of adult head and neck soft tissue sarcomas from 1990 to 2005. Thirty-six patients were seen, of whom 24 were treated at this unit, the remainder only receiving advice. The median age of the patients was 46 years. Most of the sarcomas were deep and of high or intermediate grade with a median size of 5.5 cm. Eleven different histological subtypes were identified. Wide excision was possible only in 21% of the cases. 42% of the patients developed local recurrence and 42% developed metastatic disease usually in the lungs. Overall survival was 49% at 5 years. Tumour size was the most important prognostic factor. Adult head and neck soft tissue sarcomas have a high mortality rate with a high risk of local recurrence and metastatic disease. The rarity of the disease would suggest that centralisation of care could lead to increased expertise and better outcomes.

## 1. INTRODUCTION

Soft tissue sarcomas of the head and neck are rare mesenchymal malignant neoplasms accounting for less than 10% of all soft tissue sarcomas and approximately 1% of all head and neck neoplasms [1–5]. Nevertheless, they represent an important group of tumours and are associated with significant morbidity and mortality.

There are several histological subtypes of sarcomas which present with a variety of clinical characteristics and many often require treatment with combination of surgery, radiotherapy, and chemotherapy. They are best treated in specialist sarcoma units where expert multidisciplinary approach to management is possible. In the UK, surgical management of soft tissue sarcomas in the head and neck region is undertaken by otolaryngologists, maxillofacial surgeons, as well as by musculoskeletal sarcoma surgeons, depending on the location of the lesion.

Reflecting the rarity of the disease, there is currently a scarcity of studies in the literature and, to our knowledge, there is only one published study that is based in a UK hospital in the last 15 years [4]. Most of the series which are published have reported outcome over a number of decades possibly to compensate for the rarity of the disease and to increase the size of the study sample for meaningful statistical analysis [6]. However, the spanning of studies over several decades has an important drawback in that changes in the management of sarcomas and their outcome are not always accurately reflected.

We report on our experience of the management of adult head and neck soft tissue sarcomas presenting to a regional sarcoma centre based at an orthopaedic hospital over the past 20 years.

## 2. METHODS

We have prospectively collected patient, tumour, treatment, and outcome data on all patients with bone and soft tissue sarcomas for over 20 years at the Royal Orthopaedic Hospital (Birmingham, UK). We have identified all patients with soft tissue sarcomas in the head and neck, defined as sites above the level of the clavicle. We have included all patients seen at the unit between 1990 and 2005.

We have made a number of observations related to patient demographics, tumour variables, treatment modalities, outcome, and follow up for patients with head and neck soft tissue sarcomas. Tumours were classified as deep if they were deep to the investing fascia whilst they were superficial if they lay purely in the subcutaneous tissues. The margins of excision were classified according the method of Enneking [7] with a wide margin being one in which a clear layer of normal tissue lay between the tumour and the excision margin. A marginal excision was when the excision plane passed through the reactive zone around the tumour (*clear but close*) and an intralesional excision was when tumour was incised at any part of the operation, even if a subsequent wide excision was achieved. Survival was estimated using Kaplan Meier survival curves and was determined for overall 5-year survival, and the log rank method was used to analyse the influence of various prognostic factors on survival of the patients. For situations where no events had arisen in one subgroup, chi-square testing was used to assess possible significance. Institutional approval for this study was obtained.

## 3. RESULTS

### 3.1. Patients

A total of 36 patients with head and neck soft tissue sarcomas were seen during the study period, 24 of whom were treated at this unit. This is approximately 2% of the total 1912 cases of all soft tissue sarcomas seen at this unit during the same period. The median age of the patients was 46 years with a range from 16 to 83 years. There were 24 male and 12 female patients (M : F = 2 : 1).

Thirty-five of the sarcomas were located in the neck and one was located in the scalp. The average duration of symptoms experienced by patients prior to diagnosis was 54 weeks (range 1–416 weeks) and the most commonly reported symptom was the presence of a painless lump.

The rest of the observations and analysis are based on the 24 patients who received treatment at this unit. We have excluded 12 patients who were either referred for advice only (9 patients) and received definitive treatment elsewhere or who presented with local recurrence and/or metastases after previous failed treatment (3 patients). Various observations on tumours, treatment, local control, and outcome are summarised in Table 1.

### 3.2. Tumours

The median tumour size was 5.5 cm (range 0.6–13 cm) at diagnosis. 18 of the sarcomas (75%) were deep to the investing fascia, the rest superficial to the fascia. Two of the sarcomas were low grade (18%), the rest intermediate (42%, *n* = 10) or high grades (50%, *n* = 12). All tumours which were located deep to the fascia were of high or intermediate grade. Only one patient (with a Ewing's sarcoma) had lung metastases at the time of presentation.

Eleven different histological subtypes were identified, of which malignant peripheral nerve sheath tumour (MPNST) was the commonest subtype (25%, *n* = 6). The histological subtypes are listed in Table 2.

### 3.3. Treatment

The principles of treatment used at the Unit during this time consisted of planned wide local excision followed by radiotherapy for all high-grade tumours >5 cm or where there were close margins of excision.

Eight patients were treated with surgery alone as initial treatment for their sarcomas. 13 patients had surgery and postoperative radiotherapy. Two of the three patients who had Ewing's sarcoma received neoadjuvant chemotherapy, surgery, and postoperative radiotherapy; and one received chemotherapy and surgery. The documented margins of excision were intralesional in 10 (42%), marginal in 9 (37%), and wide in 5 (21%).

### 3.4. Local control

The patients were followed up for an average period of 50 months. Local recurrence arose in 10 patients (42%) at a median time of 14 months (range 5–96 months) following initial treatment. One of the two patients with a low-grade superficial sarcoma developed local recurrence and 9 of the 22 with high- or intermediate-grade sarcomas developed local recurrence. Local recurrence was strongly related to margins achieved, arising in six of the ten with an intralesional margin (60%), three of the nine with a marginal margin (33%), and one of the five with a wide margin (20%).

Of the ten patients who developed local recurrence, four were either known to have systemic metastases already or were found to have synchronous metastases at the time of restaging. All four received palliative treatment and all died at a median of 9 months from diagnosis of the local recurrence. Six patients with local recurrence had no evidence of metastases when they developed the local recurrence and were treated aggressively with further surgical excision and radiotherapy when possible, often requiring extensive surgical reconstructions in order to obtain wide margins of excision. Two of these patients subsequently developed metastases 6 and 12 months later, respectively, and both subsequently died. The other four patients remained disease-free at a mean of 52 months following their local recurrence.

### 3.5. Metastases

Ten patients (42%) developed metastatic disease at a median time of 17 months (range 0–139 months). Eight patients developed lung metastases and two lymph node metastases. All of the patients with concomitant or previous local recurrence subsequently died as did two of the others without local recurrence. Two patients underwent surgical resection of lung metastases and remained alive and disease-free at a median of 17 months.

### 3.6. Survival

**Figure 1:**
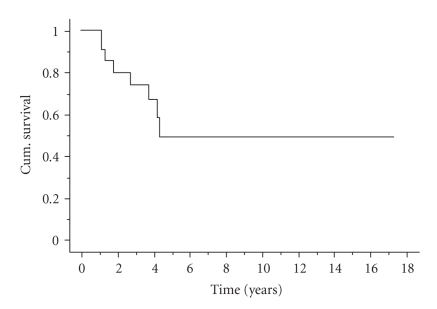
Overall 5-year survival.

Eight patients died at a median time of 2.3 years from diagnosis. Overall survival was 48.6% at five years but with wide confidence limits (plus or minus 13%) (Figure 1). We investigated the following factors for possible significance on survival.

**Figure 2:**
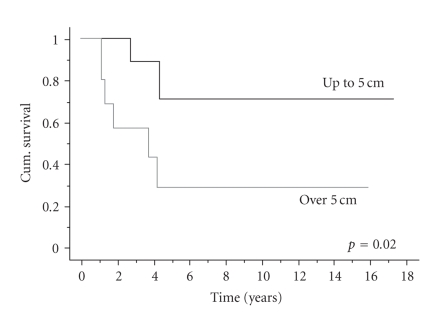
Overall survival by size of sarcoma.

- *Size.* Only 1 patient with a primary sarcoma >5 cm has yet survived more than 5 years whilst the survival for patients with tumours ≤5 cm was 71% at 5 years ( *p* = 0.02) (Figure 2).
- *Grade.* Neither of the two patients with low-grade tumours died but 8 of the 22 with high- or intermediate-grade tumours died ( *P* = .29) (chi-square).
- *Depth.* None of the 6 patients with subcutaneous sarcomas died but 8 of the 18 with deep tumours died (*P* = .0455) (chi-square).
- *Margin.* Six of the 10 patients with intralesional and two of the 9 patients with a marginal surgical margin died, however none of the 5 patients with a wide surgical margin died ( *P* = .04).
- *Age.* We could find no effect of age on survival.

Combining these factors revealed that all deaths arose in the patients with high- or intermediate-grade deep tumours. When further stratified by size, this showed that two of the seven patients with high-grade deep tumours ≤5 cm died compared with 6 of the 11 with high-grade deep tumours >5 cm ( *P* = .04).

## 4. DISCUSSION

Head and neck sarcomas are rare and the paucity of studies about their management and outcome testifies to this. Head and neck lumps are common and have a variety of diagnoses [2, 3]. Early detection and diagnosis is clearly essential. The National Institute for Health and Clinical Excellence (NICE) guidance 2005 to all UK general practitioners emphasises that “*In patients with an unexplained lump in the neck which has recently appeared or a lump which has not been diagnosed before that has changed over a period of 3 to 6 weeks, an urgent referral should be made”* [8]. Whilst more general advice is also given about lumps elsewhere in the body: “*In patients presenting with a palpable lump, an urgent referral for suspicion of soft tissue sarcoma should be made if the lump is*

- *greater than about* 5 *cm in diameter,*
- *deep to fascia, fixed, or immobile,*
- *painful,*
- *increasing in size,*
- *a recurrence after previous excision.”*

Our unit is a musculoskeletal unit that takes referrals of patients with both proven or suspected sarcomas. In the case of the head and neck tumours, some were referred directly to us for investigation and diagnosis whilst others were referred after a biopsy or imaging had confirmed the diagnosis of a sarcoma. We treated patients with sarcomas that were confined to the superficial tissues or deep muscles of the head and neck and we have not included patients with soft tissue sarcomas involving the facial skeleton or the oropharynx which pose even greater challenges in treatment [6].

Our management policy was based on principles used in treating soft tissue sarcomas at other sites. The head and neck poses particular problems however because of the proximity of so many important structures and the near impossibility of obtaining wide surgical margins in many cases. Unlike limb soft tissue sarcomas, there is no fallback option of doing an amputation if local recurrence arises.

The local recurrence rates for high-grade soft tissue sarcomas after surgical excision have been reported to be as high as 50% in the literature [3, 9, 10]. 42% of patients developed local recurrence in our study, most arising within 2 years of treatment. Barker et al. in their study reported the median time to local recurrence after treatment with surgery and/or radiotherapy to be 4 months, and Kraus et al. reported that patients who developed local recurrence did so within 3 years [11, 12].

The risk of local recurrence was higher with intralesional or marginal surgical margins as has been shown by other authors [2, 12]. In view of this, every effort should be made to maximise the margins that can be achieved at the time of the first surgical procedure, if necessary by going back and doing a further wide excision if the initial margins prove positive. All patients should have their case discussed at a multidisciplinary team meeting and the option of radiotherapy considered to try and decrease the risk of local recurrence. Even if patients do develop local recurrence, their case is not hopeless and further excision should be considered. Clearly, however, initial wide surgical margins should always be aimed for.

The commonest site for metastases was the lungs which was also the commonest cause of death. Mendenhall et al. [3] suggested that patients should undergo a chest CT before treatment and also suggested that in the absence of pulmonary metastases, other distant metastases are highly unlikely. We concur with this.

The 5-year survival rate of 49% in our study (Figure 1) is comparable to a previous UK study by Eeles et al. [4] based at the Royal Marsden Hospital of London. They analysed 103 cases seen over 44 years between 1944 and 1988 and reported 50% overall 5-year survival rate. This is similar to the results reported by Bentz et al. [6] from Memorial Sloan Kettering Cancer Centre. Most authors agree that the same prognostic factors apply to sarcomas no matter where they arise—grade, size, and depth. In the head and neck, however, local recurrence has more sinister portents because of the difficulty of subsequent management [5, 13–18].

Obtaining local control is paramount in managing these head and neck sarcomas. Obtaining wide margins may often require a multidisciplinary team consisting of a sarcoma surgeon, a head and neck surgeon and a reconstructive surgeon. A clinical oncologist is an essential part of the team to advise about radiotherapy usage. A metaanalysis published in Lancet revealed that chemotherapy did not produce a survival benefit in the treatment of soft tissue sarcomas [19]. The same analysis did, however, show a 10% benefit of chemotherapy on recurrence-free survival. Adjuvant chemotherapy is not usually advocated for localised soft tissue sarcomas but can be considered for metastatic disease as a palliative treatment.

Recent guidance from NICE, UK [20] in the management of patients with sarcomas has highlighted the importance of referring all patients with soft tissue sarcomas to a sarcoma centre where they can be managed by a multidisciplinary team (MDT). The guidance has also emphasised the importance of close collaboration between these sarcoma MDTs and site-specific head and neck surgeons and oncologists. This has been emphasised by the recent paper of Harb et al. [21]. We recommend that all surgeons who identify a suspected or proven soft tissue sarcoma of the head and neck should refer that patient to a sarcoma MDT and that all head and neck cancer MDTs should have close links with the local sarcoma MDT for management of these cases.

## Figures and Tables

**Table 1:** Patient age, tumour factors, treatment, local recurrence, and outcome. MPNST: malignant peripheral nerve sheath tumour, DFSP: dermatofibrosarcoma protuberans, MFH: malignant fibrous histiocytoma, SEF: sclerosing epithelioid fibrosarcoma, CT: chemotherapy, RT: radiotherapy.

Case no.	Age (yrs)	Diagnosis	Size (cm)	Depth	Trojani grade	Definitive treatment	Surgical margin	Local recurrence (months)	Outcome (months)
1	38	Leiomyosarcoma	0.6	Subcutaneous	High	Excision	Wide		Alive, 45
2	22	Ewing's sarcoma	1.5	Deep	High	CT + excision	Wide		Alive, 208
3	41	DFSP	1.7	Subcutaneous	Intermediate	Excision	Marginal		Alive, 20
4	24	DFSP	2	Subcutaneous	Low	Excision	Wide		Alive, 37
5	19	MPNST	3	Subcutaneous	Intermediate	Excision	Wide		Alive, 36
6	20	Ewing's sarcoma	4	Deep	High	CT + excision + RT	Intralesional		Alive, 74
7	41	Spindle cell sarcoma	4	Deep	Intermediate	Excision	Intralesional	8	Alive, 21
8	64	Spindle cell sarcoma	4	Deep	Intermediate	Excision + RT	Marginal	16	Died, 33
9	39	MPNST	5	Deep	High	Excision + RT	Intralesional	26	Died, 52
10	53	SEF	5	Deep	Intermediate	Excision	Wide	49	Alive, 139
11	62	Myxoid chondrosarcoma	5	Deep	Intermediate	Excision	Marginal	96	Alive, 157
12	77	MFH	6	Deep	High	Excision + RT	Intralesional	5	Died, 22
13	54	Myxofiibrosarcoma	6.5	Deep	High	Excision + RT	Marginal		Alive, 14
14	22	Synovial sarcoma	7	Deep	High	Excision + RT	Intralesional		Alive, 35
15	38	MPNST	7	Deep	High	Excision + RT	Intralesional		Died, 14
16	50	MPNST	7	Deep	Intermediate	Excision + RT	Marginal		Alive, 191
17	65	Liposarcoma	7	Deep	High	Excision + RT	Intralesional	12	Died, 16
18	32	MPNST	8	Deep	Intermediate	Excision + RT	Marginal		Alive, 13
19	68	Spindle cell sarcoma	9	Subcutaneous	High	Excision + RT	Wide		Alive, 12
20	73	Liposarcoma	9	Deep	High	Excision + RT	Intralesional	41	Died, 51
21	30	Ewing's sarcoma	10	Deep	High	CT + excision + RT	Intralesional	12	Died, 14
22	48	Synovial sarcoma	10	Deep	Intermediate	Excision + RT	Marginal		Alive, 11
23	65	MPNST	10	Deep	Intermediate	Excision + RT	Marginal		Died, 45
24	33	Liposarcoma	13	Subcutaneous	Low	Excision	Marginal	8	Alive, 51

**Table 2:** Histological subtypes. MPNST: malignant peripheral nerve sheath tumour, DFSP: dermatofibrosarcoma protuberans, MFH: malignant fibrous histiocytoma, SEF: sclerosing epithelioid fibrosarcoma.

Subtypes	No. of patients (%)
MPNST	6 (25%)
Ewing's sarcoma	3 (13%)
Liposarcoma	3 (13%)
Spindle cell sarcoma	3 (13%)
Synovial sarcoma	2 (8%)
DFSP	2 (8%)
Myxofibrosarcoma	1 (4%)
Leiomyosarcoma	1 (4%)
MFH	1 (4%)
Myxoid Chondrosarcoma	1 (4%)
SEF	1 (4%)
